# (*E*)-*N*-[3-(Imidazol-1-yl)-1-phenyl­propyl­idene]hydroxyl­amine

**DOI:** 10.1107/S1600536812004266

**Published:** 2012-02-10

**Authors:** Hoong-Kun Fun, Ching Kheng Quah, Mohamed I. Attia, Maha S. Almutairi, Soraya W. Ghoneim

**Affiliations:** a X-ray Crystallography Unit, School of Physics, Universiti Sains Malaysia, 11800 USM, Penang, Malaysia; b Department of Pharmaceutical Chemistry, College of Pharmacy, King Saud University, Riyadh 11451, Saudi Arabia

## Abstract

The title compound, C_12_H_13_N_3_O, exists in an *E* configuration with respect to the C=N bond [1.285 (2) Å]. The imidazole ring forms a dihedral angle of 75.97 (10)° with the phenyl ring. In the crystal, mol­ecules are linked *via* O—H⋯N and C—H⋯N hydrogen bonds into sheets lying parallel to (001). The crystal structure also features C—H⋯π inter­actions.

## Related literature

For general background to and the pharmacological activities of the title compound, see: Weinberg (1996 ▶); Wildfeuer *et al.* (1998 ▶); Georgopapadakou (1998 ▶). For standard bond-length data, see: Allen *et al.* (1987 ▶). For the stability of the temperature controller used for the data collection, see: Cosier & Glazer (1986 ▶).
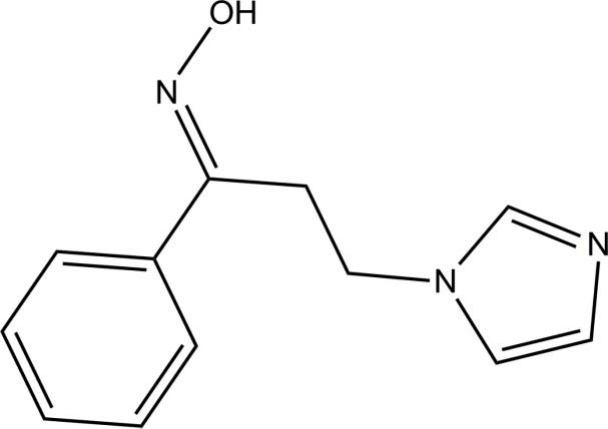


## Experimental

### 

#### Crystal data

C_12_H_13_N_3_O
*M* *_r_* = 215.25
Monoclinic, 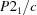

*a* = 8.0990 (1) Å
*b* = 14.0513 (2) Å
*c* = 9.9771 (2) Å
β = 93.058 (1)°
*V* = 1133.79 (3) Å^3^
*Z* = 4
Mo *K*α radiation
μ = 0.08 mm^−1^
*T* = 100 K
0.35 × 0.18 × 0.11 mm

#### Data collection

Bruker SMART APEXII CCD diffractometer
Absorption correction: multi-scan (*SADABS*; Bruker, 2009 ▶) *T*_min_ = 0.972, *T*_max_ = 0.991
12642 measured reflections
3300 independent reflections
2653 reflections with *I* > 2σ(*I*)
*R*_int_ = 0.026

#### Refinement

*R*[*F* ^2^ > 2σ(*F* ^2^)] = 0.065
*wR*(*F* ^2^) = 0.138
*S* = 1.13
3300 reflections
149 parameters
H atoms treated by a mixture of independent and constrained refinement
Δρ_max_ = 0.36 e Å^−3^
Δρ_min_ = −0.29 e Å^−3^

### 

Data collection: *APEX2* (Bruker, 2009 ▶); cell refinement: *SAINT* (Bruker, 2009 ▶); data reduction: *SAINT*; program(s) used to solve structure: *SHELXTL* (Sheldrick, 2008 ▶); program(s) used to refine structure: *SHELXTL*; molecular graphics: *SHELXTL*; software used to prepare material for publication: *SHELXTL* and *PLATON* (Spek, 2009 ▶).

## Supplementary Material

Crystal structure: contains datablock(s) global, I. DOI: 10.1107/S1600536812004266/hb6619sup1.cif

Structure factors: contains datablock(s) I. DOI: 10.1107/S1600536812004266/hb6619Isup2.hkl

Supplementary material file. DOI: 10.1107/S1600536812004266/hb6619Isup3.cml

Additional supplementary materials:  crystallographic information; 3D view; checkCIF report

## Figures and Tables

**Table 1:** Hydrogen-bond geometry (Å, °) *Cg*1 is the centroid of the C1–C6 phenyl ring.

*D*—H⋯*A*	*D*—H	H⋯*A*	*D*⋯*A*	*D*—H⋯*A*
O1—H1O1⋯N3^i^	0.90 (3)	1.82 (3)	2.712 (2)	176 (3)
C2—H2*A*⋯N1^ii^	0.95	2.56	3.477 (2)	162
C12—H12*A*⋯*Cg*1^iii^	0.95	2.74	3.558 (2)	145

Symmetry codes: (i) 
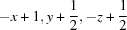
; (ii) 
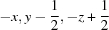
; (iii) 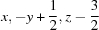.

**Fig. 1.**
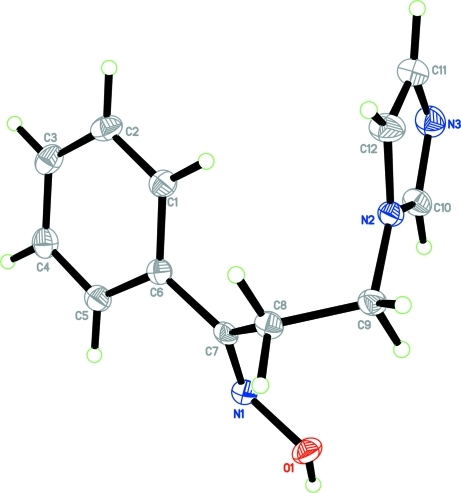
The molecular structure of the title compound showing 50% probability displacement ellipsoids for non-H atoms.

**Fig. 2.**
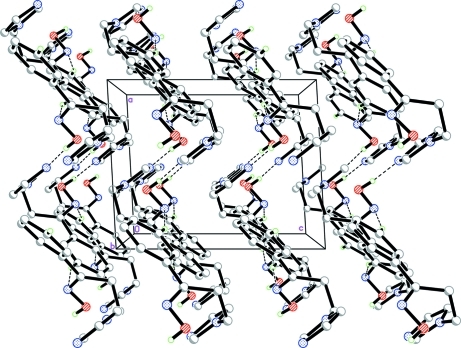
The crystal structure of the title compound, viewed along the b axis. H atoms not involved in hydrogen bonds (dashed lines) have been omitted for clarity.

## supplementary crystallographic information

### Comment

A significant increase in fungal infections has been observed over the past three decades. Many reports of invasive topical and systemic infections caused by the opportunistic pathogen Candida species are always associated with the use of broad-spectrum antibiotics, immunosuppressive agents, anticancer and anti-AIDS drugs (Weinberg, 1996). One of the major problems in the treatment of Candida infections is the spread of antifungal drug resistance mainly in patients chronically subjected to antimycotic therapy such as HIV-infected patients (Wildfeuer *et al.*, 1998; Georgopapadakou, 1998). Azoles are commonly used as antifungal agent specially for Candida infections as many marketed drugs contain the azole moiety. The title compound contains the azole moiety and it was prepared to test its antifungal potential and will be further elaborated to other azole-containing new bioactive chemical entities.

In the title compound, Fig.1, the imidazole ring (N2/N3/C10-C12, maximum deviation of 0.001 (2) Å at atoms N3, C11 and C12) forms a dihedral angle of 75.97 (10)° with the phenyl ring (C1-C6). Bond lengths (Allen *et al.*, 1987) and angles are within normal ranges. The title compound exists in *trans* configuration with respect to the C7═N1 bond [1.285 (2) Å].

In the crystal structure, Fig. 2, molecules are linked *via* intermolecular O1–H1O1···N3 and C2–H2A···N1 hydrogen bonds (Table 1) into two-dimensional networks parallel to (001). The crystal structure is further consolidated by C12–H12A···Cg1^iii^ (Table 1) interactions, where Cg1 is the centroid of C1-C6 phenyl ring.

### Experimental

A mixture of 3-(1*H*-imidazol-1-yl)-1-phenylpropan-1-one (0.02 g, 0.1 mmol), hydroxylamine hydrochloride (0.14 g, 0.2 mol), and KOH (0.112 g, 0.2 mmol) in ethanol (10 ml) was refluxed under stirring for 18h. The reaction mixture was allowed to cool to room temperature and the insolubles were removed by filtration. The filtrate was evaporated under vacuum and the residue was suspended in water (10 ml), filtered, dried and recrystallized from ethanol to yield colourless blocks of the title compound.

### Refinement

Atom H1O1 was located in a difference Fourier map and refined freely with O1-H1O1 = 0.90 (3) Å. The remaining H atoms were positioned geometrically and refined using a riding model with C–H = 0.95 or 0.99 Å and *U*_iso_(H) = 1.2 *U*_eq_(C).

### Crystal data

**Table:**

C_12_H_13_N_3_O	*F*(000) = 456
*M**_r_* = 215.25	*D*_x_ = 1.261 Mg m^−^^3^
Monoclinic, *P*2_1_/*c*	Mo *K*α radiation, λ = 0.71073 Å
Hall symbol: -P 2ybc	Cell parameters from 5276 reflections
*a* = 8.0990 (1) Å	θ = 2.5–30.1°
*b* = 14.0513 (2) Å	µ = 0.08 mm^−^^1^
*c* = 9.9771 (2) Å	*T* = 100 K
β = 93.058 (1)°	Block, colourless
*V* = 1133.79 (3) Å^3^	0.35 × 0.18 × 0.11 mm
*Z* = 4	

### Data collection

**Table:**

Bruker SMART APEXII CCD diffractometer	3300 independent reflections
Radiation source: fine-focus sealed tube	2653 reflections with *I* > 2σ(*I*)
Graphite monochromator	*R*_int_ = 0.026
φ and ω scans	θ_max_ = 30.2°, θ_min_ = 2.5°
Absorption correction: multi-scan (*SADABS*; Bruker, 2009)	*h* = −9→11
*T*_min_ = 0.972, *T*_max_ = 0.991	*k* = −18→19
12642 measured reflections	*l* = −14→14

### Refinement

**Table:**

Refinement on *F*^2^	Primary atom site location: structure-invariant direct methods
Least-squares matrix: full	Secondary atom site location: difference Fourier map
*R*[*F*^2^ > 2σ(*F*^2^)] = 0.065	Hydrogen site location: inferred from neighbouring sites
*wR*(*F*^2^) = 0.138	H atoms treated by a mixture of independent and constrained refinement
*S* = 1.13	*w* = 1/[σ^2^(*F*_o_^2^) + (0.0228*P*)^2^ + 1.5032*P*] where *P* = (*F*_o_^2^ + 2*F*_c_^2^)/3
3300 reflections	(Δ/σ)_max_ = 0.001
149 parameters	Δρ_max_ = 0.36 e Å^−^^3^
0 restraints	Δρ_min_ = −0.29 e Å^−^^3^

### Special details

**Table:**

Experimental. The crystal was placed in the cold stream of an Oxford Cryosystems Cobra open-flow nitrogen cryostat (Cosier & Glazer, 1986) operating at 100.0 (1) K.
Geometry. All esds (except the esd in the dihedral angle between two l.s. planes) are estimated using the full covariance matrix. The cell esds are taken into account individually in the estimation of esds in distances, angles and torsion angles; correlations between esds in cell parameters are only used when they are defined by crystal symmetry. An approximate (isotropic) treatment of cell esds is used for estimating esds involving l.s. planes.
Refinement. Refinement of F^2^ against ALL reflections. The weighted R-factor wR and goodness of fit S are based on F^2^, conventional R-factors R are based on F, with F set to zero for negative F^2^. The threshold expression of F^2^ > 2sigma(F^2^) is used only for calculating R-factors(gt) etc. and is not relevant to the choice of reflections for refinement. R-factors based on F^2^ are statistically about twice as large as those based on F, and R- factors based on ALL data will be even larger.

### Fractional atomic coordinates and isotropic or equivalent isotropic displacement parameters (Å^2^)

**Table:**

	*x*	*y*	*z*	*U*_iso_*/*U*_eq_	
O1	0.35159 (18)	1.05298 (10)	0.16643 (14)	0.0222 (3)	
N1	0.2389 (2)	1.00261 (11)	0.24156 (16)	0.0190 (3)	
N2	0.37457 (19)	0.78810 (11)	0.03621 (15)	0.0155 (3)	
N3	0.4790 (2)	0.66854 (12)	0.15734 (17)	0.0242 (4)	
C1	−0.0041 (2)	0.79271 (13)	0.20420 (18)	0.0185 (4)	
H1A	0.0470	0.7650	0.1301	0.022*	
C2	−0.1123 (2)	0.73889 (14)	0.27705 (19)	0.0212 (4)	
H2A	−0.1339	0.6745	0.2529	0.025*	
C3	−0.1888 (2)	0.77890 (14)	0.38485 (19)	0.0223 (4)	
H3A	−0.2612	0.7417	0.4354	0.027*	
C4	−0.1592 (2)	0.87348 (14)	0.41854 (19)	0.0215 (4)	
H4A	−0.2131	0.9014	0.4911	0.026*	
C5	−0.0509 (2)	0.92730 (14)	0.34644 (19)	0.0195 (4)	
H5A	−0.0313	0.9920	0.3700	0.023*	
C6	0.0299 (2)	0.88731 (13)	0.23934 (18)	0.0154 (3)	
C7	0.1555 (2)	0.94228 (12)	0.16866 (17)	0.0144 (3)	
C8	0.1779 (2)	0.92702 (13)	0.02083 (18)	0.0164 (4)	
H8A	0.1631	0.9888	−0.0259	0.020*	
H8B	0.0896	0.8838	−0.0150	0.020*	
C9	0.3457 (2)	0.88532 (13)	−0.01285 (18)	0.0177 (4)	
H9A	0.3536	0.8858	−0.1115	0.021*	
H9B	0.4344	0.9270	0.0261	0.021*	
C10	0.4732 (2)	0.76180 (14)	0.14318 (19)	0.0197 (4)	
H10A	0.5315	0.8053	0.2013	0.024*	
C11	0.3783 (3)	0.63328 (14)	0.0532 (2)	0.0256 (4)	
H11A	0.3577	0.5677	0.0365	0.031*	
C12	0.3129 (3)	0.70599 (14)	−0.0221 (2)	0.0233 (4)	
H12A	0.2396	0.7011	−0.0994	0.028*	
H1O1	0.403 (3)	1.0915 (19)	0.227 (3)	0.037 (7)*	

### Atomic displacement parameters (Å^2^)

**Table:**

	*U*^11^	*U*^22^	*U*^33^	*U*^12^	*U*^13^	*U*^23^
O1	0.0233 (7)	0.0180 (7)	0.0258 (7)	−0.0076 (6)	0.0066 (6)	−0.0048 (6)
N1	0.0181 (8)	0.0159 (7)	0.0232 (8)	−0.0006 (6)	0.0033 (6)	−0.0019 (6)
N2	0.0154 (7)	0.0140 (7)	0.0169 (7)	0.0004 (6)	0.0004 (6)	−0.0012 (6)
N3	0.0239 (9)	0.0225 (9)	0.0261 (9)	0.0041 (7)	0.0016 (7)	0.0042 (7)
C1	0.0178 (9)	0.0193 (9)	0.0181 (8)	−0.0008 (7)	−0.0018 (7)	−0.0015 (7)
C2	0.0223 (9)	0.0185 (9)	0.0221 (9)	−0.0049 (7)	−0.0049 (7)	0.0010 (7)
C3	0.0201 (9)	0.0263 (10)	0.0204 (9)	−0.0036 (8)	0.0003 (7)	0.0064 (8)
C4	0.0199 (9)	0.0263 (10)	0.0185 (9)	0.0017 (8)	0.0023 (7)	0.0015 (7)
C5	0.0195 (9)	0.0184 (9)	0.0205 (9)	0.0012 (7)	−0.0001 (7)	−0.0014 (7)
C6	0.0143 (8)	0.0163 (8)	0.0149 (8)	0.0010 (7)	−0.0041 (6)	0.0017 (6)
C7	0.0145 (8)	0.0121 (8)	0.0163 (8)	0.0038 (6)	−0.0013 (6)	0.0021 (6)
C8	0.0176 (9)	0.0160 (8)	0.0152 (8)	0.0024 (7)	−0.0021 (7)	0.0029 (6)
C9	0.0205 (9)	0.0148 (8)	0.0179 (8)	0.0014 (7)	0.0030 (7)	0.0021 (7)
C10	0.0207 (9)	0.0190 (9)	0.0191 (9)	0.0026 (7)	−0.0017 (7)	−0.0009 (7)
C11	0.0258 (10)	0.0177 (9)	0.0338 (11)	−0.0002 (8)	0.0057 (9)	−0.0026 (8)
C12	0.0252 (10)	0.0197 (9)	0.0244 (9)	−0.0019 (8)	−0.0044 (8)	−0.0043 (8)

### Geometric parameters (Å, º)

**Table:**

O1—N1	1.403 (2)	C4—C5	1.388 (3)
O1—H1O1	0.90 (3)	C4—H4A	0.9500
N1—C7	1.285 (2)	C5—C6	1.400 (3)
N2—C10	1.350 (2)	C5—H5A	0.9500
N2—C12	1.374 (2)	C6—C7	1.485 (3)
N2—C9	1.466 (2)	C7—C8	1.511 (2)
N3—C10	1.319 (3)	C8—C9	1.534 (3)
N3—C11	1.378 (3)	C8—H8A	0.9900
C1—C2	1.391 (3)	C8—H8B	0.9900
C1—C6	1.398 (3)	C9—H9A	0.9900
C1—H1A	0.9500	C9—H9B	0.9900
C2—C3	1.389 (3)	C10—H10A	0.9500
C2—H2A	0.9500	C11—C12	1.359 (3)
C3—C4	1.389 (3)	C11—H11A	0.9500
C3—H3A	0.9500	C12—H12A	0.9500
			
N1—O1—H1O1	103.3 (17)	N1—C7—C6	115.28 (16)
C7—N1—O1	111.57 (15)	N1—C7—C8	124.02 (17)
C10—N2—C12	106.95 (16)	C6—C7—C8	120.70 (15)
C10—N2—C9	126.60 (15)	C7—C8—C9	114.95 (15)
C12—N2—C9	126.38 (16)	C7—C8—H8A	108.5
C10—N3—C11	105.09 (17)	C9—C8—H8A	108.5
C2—C1—C6	120.45 (18)	C7—C8—H8B	108.5
C2—C1—H1A	119.8	C9—C8—H8B	108.5
C6—C1—H1A	119.8	H8A—C8—H8B	107.5
C3—C2—C1	120.27 (18)	N2—C9—C8	114.23 (15)
C3—C2—H2A	119.9	N2—C9—H9A	108.7
C1—C2—H2A	119.9	C8—C9—H9A	108.7
C4—C3—C2	119.75 (18)	N2—C9—H9B	108.7
C4—C3—H3A	120.1	C8—C9—H9B	108.7
C2—C3—H3A	120.1	H9A—C9—H9B	107.6
C5—C4—C3	120.14 (18)	N3—C10—N2	111.89 (17)
C5—C4—H4A	119.9	N3—C10—H10A	124.1
C3—C4—H4A	119.9	N2—C10—H10A	124.1
C4—C5—C6	120.72 (18)	C12—C11—N3	110.14 (18)
C4—C5—H5A	119.6	C12—C11—H11A	124.9
C6—C5—H5A	119.6	N3—C11—H11A	124.9
C1—C6—C5	118.63 (17)	C11—C12—N2	105.94 (17)
C1—C6—C7	120.40 (16)	C11—C12—H12A	127.0
C5—C6—C7	120.90 (16)	N2—C12—H12A	127.0
			
C6—C1—C2—C3	0.5 (3)	C5—C6—C7—C8	−147.91 (17)
C1—C2—C3—C4	1.1 (3)	N1—C7—C8—C9	66.1 (2)
C2—C3—C4—C5	−1.3 (3)	C6—C7—C8—C9	−114.85 (18)
C3—C4—C5—C6	−0.1 (3)	C10—N2—C9—C8	−104.3 (2)
C2—C1—C6—C5	−1.9 (3)	C12—N2—C9—C8	79.3 (2)
C2—C1—C6—C7	175.03 (17)	C7—C8—C9—N2	65.6 (2)
C4—C5—C6—C1	1.7 (3)	C11—N3—C10—N2	0.1 (2)
C4—C5—C6—C7	−175.24 (17)	C12—N2—C10—N3	0.0 (2)
O1—N1—C7—C6	−178.52 (14)	C9—N2—C10—N3	−176.95 (17)
O1—N1—C7—C8	0.6 (2)	C10—N3—C11—C12	−0.1 (2)
C1—C6—C7—N1	−145.59 (17)	N3—C11—C12—N2	0.1 (2)
C5—C6—C7—N1	31.3 (2)	C10—N2—C12—C11	−0.1 (2)
C1—C6—C7—C8	35.2 (2)	C9—N2—C12—C11	176.87 (18)

### Hydrogen-bond geometry (Å, º)

Cg1 is the centroid of the C1–C6 phenyl ring.

**Table:**

*D*—H···*A*	*D*—H	H···*A*	*D*···*A*	*D*—H···*A*
O1—H1*O*1···N3^i^	0.90 (3)	1.82 (3)	2.712 (2)	176 (3)
C2—H2*A*···N1^ii^	0.95	2.56	3.477 (2)	162
C12—H12*A*···*Cg*1^iii^	0.95	2.74	3.558 (2)	145

Symmetry codes: (i) −*x*+1, *y*+1/2, −*z*+1/2; (ii) −*x*, *y*−1/2, −*z*+1/2; (iii) *x*, −*y*+1/2, *z*−3/2.
